# Design and function of indoor versus regular custom-made footwear in people with diabetes at high ulcer risk

**DOI:** 10.1080/19424280.2025.2491023

**Published:** 2025-06-20

**Authors:** Lisa E. Vossen, Jaap J. Van Netten, Sicco A. Bus

**Affiliations:** a Department of Rehabilitation Medicine, Amsterdam UMC, University of Amsterdam, Amsterdam, Netherlands; b Amsterdam Movement Sciences, Rehabilitation & Development, Amsterdam, Netherlands

**Keywords:** Diabetic foot, footwear, mechanical stress, design, adherence

## Introduction

Custom-made footwear specifically designed to use indoors increases adherence to prescription footwear in people with diabetes, peripheral neuropathy, and high ulcer risk, compared to using only regular custom-made footwear (Keukenkamp et al., 2022). The design and biomechanical function of such footwear are important if they are to replace regular footwear for indoor use. A pilot study showed similar maximum peak pressures with regular footwear. A more in-depth analysis of the pressure distribution and center-of-pressure parameters for the stability of this indoor footwear in a representative sample of subjects is yet to be conducted. Such investigation can demonstrate if indoor footwear is a biomechanically safe replacement for regular custom-made footwear for indoor use.

## Purpose of the study

The aim was to compare indoor and regular custom-made footwear for design, pressure distribution, and gait stability in people with diabetes at high risk of ulceration.

## Methods

Custom-made indoor footwear was provided to 36 participants with diabetes, peripheral neuropathy, and a recently healed plantar foot ulcer or (partial) foot amputation. All participants were in possession of regular pressure-optimized custom-made footwear and the indoor footwear was designed based on the last of the regular footwear. The indoor footwear was lighter in weight, easier to don and doff, more comfortable because of the use of softer materials for the shoe upper, and cheaper to produce, all aspects that should facilitate indoor use. Both footwear types were assessed for shoe design elements and agreement in design was calculated using Cohen’s Kappa and percentage agreement. In-shoe plantar pressures were measured during walking in both shoes using Pedar-X, from which multiple peak plantar pressure and center-of-pressure parameters were calculated. Peak pressure parameters, including multidimensional parameters (Vossen et al., 2025), were used to define offloading effectiveness, while center-of-pressure parameters were used to define foot roll-over and gait stability (Menz et al., 2018; Pol et al., 2021). Scalar peak pressure parameters between footwear types were assessed using paired *t*-tests. Peak pressure distributions in spatial and temporal domains and center-of-pressure parameters were assessed using paired *t*-tests and statistical parametric mapping.

## Results

**Figure 1.**
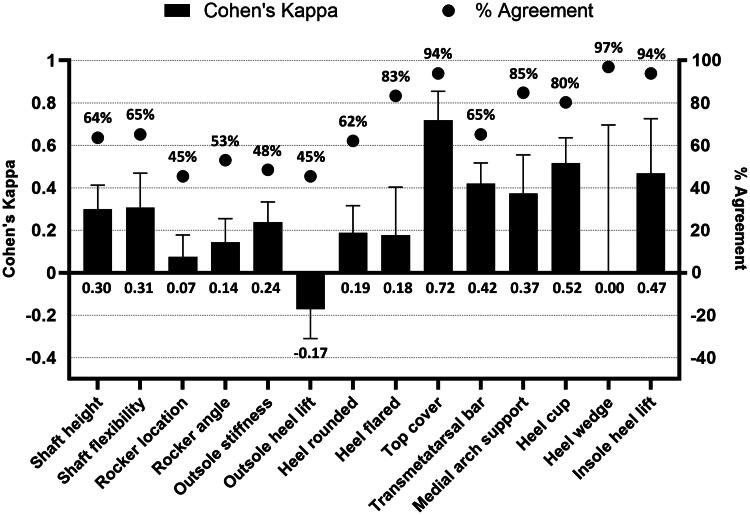
Cohen’s Kappa agreement analysis for each of the design elements. The strength of Cohen’s Kappa can be interpreted as follows: <0 poor, 0–0.20 slight, 0.21–0.4 fair, 0.41–0.60 moderate, 0.61–0.8 substantial, >0.81 almost perfect (Landis & Koch, 1977). The percentage of agreement is shown in dots.

**Figure 2.**
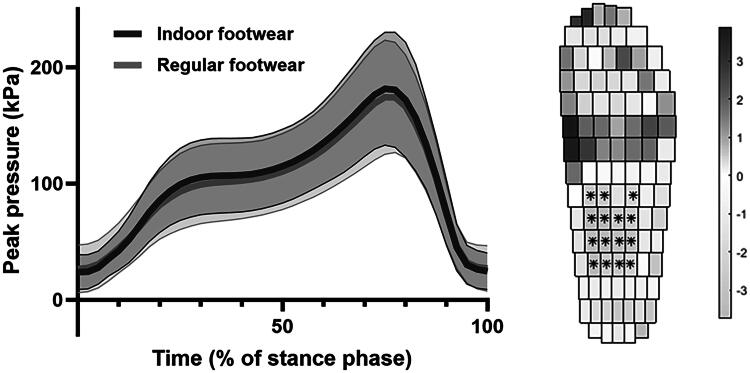
Peak plantar pressure in temporal (left) and spatial (right) parameters. Spatial results are shown as the statistical differences, with significant differences indicated with an asterisk. Positive values indicate higher values for the indoor footwear, and negative values for the regular footwear.

A total of 132 shoes, 66 per footwear type, of 36 participants were analyzed. Cohen’s Kappa for agreement on design elements ranged from −0.17 to 0.72 and percentage agreement from 45% to 97% (Figure 1). Outcomes for all peak pressure parameters were non-significantly higher in the indoor compared to regular footwear (*p* > 0.05). The maximum average difference in the forefoot regions was 10.1 kPa for maximum peak pressure, 1.22 kPa.s for pressure time integral, and 0.13 kPa/mm for the pressure gradient. Overall pressure distribution in spatial and temporal dimensions for all participants did not significantly differ in the forefoot area (Figure 2). Center-of-pressure parameters, for both foot roll-over and stability, were not significantly different between footwear types.

## Discussion and conclusion

No significant differences were found in pressure distribution, foot roll-over, and gait stability between footwear types, despite differences found in footwear design. These results show that custom-made indoor footwear is a biomechanically safe alternative for use indoors to regular custom-made footwear in people with diabetes at high risk for foot ulceration.
